# Chemotaxis of *Burkholderia *sp. Strain SJ98 towards chloronitroaromatic compounds that it can metabolise

**DOI:** 10.1186/1471-2180-12-19

**Published:** 2012-02-01

**Authors:** Janmejay Pandey, Narinder K Sharma, Fazlurrahman Khan, Anuradha Ghosh, John G Oakeshott, Rakesh K Jain, Gunjan Pandey

**Affiliations:** 1 Institute of Microbial Technology, Sector 39A, Chandigarh 160036, India; 2 Georgia Health Science University, Augusta GA 30912, USA; 3 Kansas State University, Manhattan, KS 66506, USA; 4 CSIRO Ecosystem Sciences, GPO Box 1700, Canberra ACT 2601, Australia

## Abstract

**Background:**

*Burkholderia *sp. strain SJ98 is known for its chemotaxis towards nitroaromatic compounds (NACs) that are either utilized as sole sources of carbon and energy or co-metabolized in the presence of alternative carbon sources. Here we test for the chemotaxis of this strain towards six chloro-nitroaromatic compounds (CNACs), namely 2-chloro-4-nitrophenol (2C4NP), 2-chloro-3-nitrophenol (2C3NP), 4-chloro-2-nitrophenol (4C2NP), 2-chloro-4-nitrobenzoate (2C4NB), 4-chloro-2-nitrobenzoate (4C2NB) and 5-chloro-2-nitrobenzoate (5C2NB), and examine its relationship to the degradation of such compounds.

**Results:**

Strain SJ98 could mineralize 2C4NP, 4C2NB and 5C2NB, and co-metabolically transform 2C3NP and 2C4NB in the presence of an alternative carbon source, but was unable to transform 4C2NP under these conditions. Positive chemotaxis was only observed towards the five metabolically transformed CNACs. Moreover, the chemotaxis was induced by growth in the presence of the metabolisable CNAC. It was also competitively inhibited by the presence of nitroaromatic compounds (NACs) that it could metabolise but not by succinate or aspartate.

**Conclusions:**

*Burkholderia *sp. strain SJ98 exhibits metabolic transformation of, and inducible chemotaxis towards CNACs. Its chemotactic responses towards these compounds are related to its previously demonstrated chemotaxis towards NACs that it can metabolise, but it is independently inducible from its chemotaxis towards succinate or aspartate.

## Background

Microbial bioremediation can be an efficient, economic and environmentally friendly alternative to other physico-chemical approaches used for the cleanup of contaminated soils [1-3]. However, *in situ *bioremediation trials show that this approach is not as successful under natural environmental conditions as would be expected from *in vitro *experiments [4,5]. One of the major reasons for this is the limited bioavailability of the pollutant, which in turn is a function of its hydrophobicity, solubility and persistence in the environmental matrix [4,5]. Increasingly, however, it has been recognized that microbial chemotaxis towards the pollutant can also be a major determinant [6-9].

Chloro-nitroaromatic compounds (CNACs) are a new class of toxic xenobiotic compounds that have been extensively used over the last few decades in the synthesis of pesticides, herbicides, dyes etc. Because of their stability, toxicity, mutagenicity and potential carcinogenicity, many CNACs, including chloro-nitrophenols (CNPs), chloro-nitrobenzenes (CNs) and chloro-nitrobenzoates (CNBs), have been listed as priority pollutants by organizations such as the United States Environment Protection Agency [10-13]. Microbial degradation could in theory be used to restore sites contaminated with CNACs but these compounds have proven to be extremely stable and recalcitrant to metabolic degradation [14] and there are very few reports of pure microbial isolates which are capable of degrading them [15-18].

We have recently shown that *Burkholderia *sp. strain SJ98 can degrade 2-chloro-4-nitrophenol (2C4NP) and utilize it as sole source of carbon and energy [19]. This strain was previously shown to mount a chemotactic response towards a number of nitroaromatic compounds (NACs) that it can either completely metabolize or co-metabolically transform in the presence of an alternative carbon source [20-23]. Here we show that strain SJ98 is also chemotactic towards certain CNACs which it is able to metabolise. To the best of our knowledge, this is the first report of microbial chemotaxis towards CNACs.

## Methods

### Bacterial strain, media and culture conditions

*Burkholderia *sp. SJ98 was previously isolated by a "chemotactic enrichment technique" from a pesticide-contaminated soil sample [22]. Initially this strain was identified as *Ralstonia *sp. strain SJ98 but it has now been re-classified as a *Burkholderia *sp. [24]. During the present study, strain SJ98 was grown in minimal medium (MM) supplemented with the test CNACs. CNACs were added as filter-sterilized solutions in MM to obtain working concentrations of 50-500 μM. Filter-sterilized succinate (10 mM) was added as an alternative carbon source to the MM where necessary. The composition of the medium was as described earlier [25]. Incubations were carried out at 30°C under shaking conditions (180 rpm) and growth was monitored spectrophotometrically at 600 nm. For culture maintenance, strain SJ98 was routinely grown on nutrient agar (NA) or nutrient broth (NB) prepared according to the manufacturer's recommendations and as described earlier [19].

### Metabolic activity of strain SJ98 on tested CNACs

In tandem with the chemotactic assays (see below), the metabolic activity of strain SJ98 on the tested CNACs was also determined by growth studies, resting cell assays and biochemical analyses of the growth medium to detect transformation products. The purpose of, and methods for each of these studies are indicated below:

#### Growth studies

The initial screening of the metabolic activity of strain SJ98 on test CNACs was performed with growth studies using MM supplemented with 50-500 μM of each CNAC as the sole sources of carbon and energy. Metabolic activity was determined by growth, monitored spectrophotometrically. For CNACs that could not be utilized as sole sources of carbon and energy during the initial screening, the culture medium for subsequent growth studies was supplemented with 10 mM of sodium succinate.

#### Resting cell studies

Resting cell studies were carried out to identify some of the degradation intermediates and elucidate the catabolic pathways of those CNACs that were completely mineralized by strain SJ98 (described below). These studies were performed according to procedures described earlier [19,20,26]; briefly, cells of strain SJ98 grown in 250 ml of nutrient broth (Sigma-Aldrich (GmbH, Germany)) medium up to mid-exponential phase (OD_600 _0.45-0.60) were harvested by centrifugation at 3500 rpm for 8-10 min at ambient temperature, washed twice with 10 mM sodium phosphate buffer (pH 7.2) and then re-suspended in 50 ml of MM supplemented with 300 μM of the test CNAC (2C4NP or 4C2NB) and incubated at 30°C. Induction of CNAC degradation was monitored via visible decolorization of the induction medium. (Since most CNACs are yellow colored in aqueous growth medium and turn colorless upon microbial catabolic activities, the decolorization of the culture medium is used as an important indicator for induction of the degradation mechanism). After induction, the cells were harvested, washed and re-suspended in 20 ml of MM. The re-suspension was divided into two aliquots, one of which was heat killed (boiled for 10 min) and used as the negative control, and the other of which was incubated with 300 μM of test compound at 30°C. Samples (0.5 ml of supernatant) from both aliquots were withdrawn at 10 min intervals and stored at -20°C for further analysis.

#### Chloride, nitrite and ammonia release

To obtain preliminary information about the nature (oxidative vs. reductive) of the catabolic degradation of 2C4NP and 4C2NB by strain SJ98, samples collected from the growth studies and resting cell studies were concurrently tested for Cl^-^, NO_2_^- ^and NH_4_^+ ^release. Chloride and nitrite ions were detected with spectrophotometric methods as described earlier [27,28] and quantified by reference to standard plots generated with known concentrations of NaCl and NaNO_2_. Released ammonia in the growth medium was tested with the 'Ammonia Assay Kit' from Sigma-Aldrich (GmbH, Germany) according to the manufacturer's instructions. Uninoculated growth media were used as the negative control in all cases.

### Identification of transformation products

#### Extraction and analytical methods

Culture supernatants were subjected to organic extraction according to previously published procedures [29]. Briefly, culture supernatants were extracted with an equal volume of ethyl acetate at neutral pH, the organic layer was carefully separated and the remaining aqueous phase then acidified to pH 2.0 with 5 M HCl and again extracted with an equal volume of ethyl acetate. The neutral and acidic organic layers (extracts) were pooled together, evaporated to dryness with a rotary evaporator (BUCHI-Postfach, Flawil, Switzerland) and then dissolved in 150 μl of ethyl acetate. The latter was then subjected to thin layer chromatography (TLC) and gas chromatography (GC) using standard procedures. The identity of transformation intermediates was ascertained by comparing the Rf and Rt values obtained from the TLC and GC analyses respectively to those of authentic standards. Uninoculated media were used as controls for abiotic transformation of test CNACs.

Culture supernatants were also subjected to high performance liquid chromatography (HPLC) using a Waters 600 model (Waters, Millford USA) equipped with a Waters 996 photodiode array detector. Detection of the transformation intermediates was carried out by scanning the samples at 210-390 nm. Sample separation was carried out using a Waters Spherisorb 5 μm C8 reverse phase column as the stationary phase and 1% glacial acetic acid in methanol and 1% glacial acetic acid in the ratio 80:20 at a constant flow rate of 1.0 ml.min^-1 ^as the mobile phase. The identity of peaks was established by comparison of UV-visible spectra and retention times (Rt) to those for the peaks obtained from standard compounds.

### Chemotaxis of strain SJ98 towards CNACs

The chemotactic behaviour of strain SJ98 towards test CNACs was investigated qualitatively with drop plate and swarm plate assays and quantitatively with capillary assays according to procedures described earlier [9,20,30]. Competitive capillary assays were also conducted to determine the effect of co-occurrence of potential chemotactic competitors on the chemotactic behaviour of strain SJ98 towards the CNACs.

#### Drop plate assay

Cells were grown in MM plus 10 mM glucose, MM plus the test CNAC, or MM plus both the test CNAC and 10 mM glucose. The concentration of CNACs in the growth medium was set at the optimum value (i.e., eliciting the strongest chemotactic response in the quantitative capillary assays described below). The cells were harvested at mid-log phase (OD_600 _~0.35) by centrifugation at 3500 rpm for 8-10 min. Harvested cells were washed twice with phosphate buffered saline (PBS), resuspended in drop plate assay medium (MM plus 0.3% bacto agar) and poured into 96 mm petri-plates. A few crystals of the respective compound(s) were placed in the center of petri-plates which were then incubated at 25°C. The chemotactic response was observed after 4-6 hrs of incubation. A positive response was indicated by the formation of concentric chemotaxis rings, due to bacterial cell accumulation encircling the crystals.

#### Swarm plate assay

The swarm plate assays were performed in petri-plates containing swarm plate medium (MM containing 0.2% bacto agar) supplemented with the optimal response concentration of the test CNAC. About 50-60 μl cell suspension (OD_600 _~2.0 in MM) was gently poured onto the center of the plate which was then incubated at 25°C. A chemotactic response was indicated by formation of exocentric rings after 12-16 hrs of incubation.

#### Capillary assay

Quantitation of the chemotactic response was performed using a high throughput capillary assay according to a protocol described earlier [20]. Preliminary assays tested a range of concentrations of each CNAC (from 50-500 μM in 50 μM increments) and subsequent assays were then conducted at the 'optimum' concentration of each. The chemotaxis buffer consisted of 100 mM potassium phosphate (pH 7.0) and 20 μM EDTA. A 10 μl glass capillary was filled with a solution of the test CNAC (in chemotaxis buffer) and then inserted into a glass slide containing a suspension (10^7-8 ^cells.ml^-1^) of strain SJ98 cells and incubated at 25°C for 30 min. The contents of the capillary tubes were then serially diluted and plated onto non-selective medium (nutrient agar). Colony forming units (CFUs) were counted after 48 h incubation at 30°C. The strength of chemotactic response was expressed in terms of the chemotaxis index (CI), which is the ratio of the number of CFUs produced from the capillary containing the test compound(s) to CFUs produced from a control capillary (i.e. just chemotaxis buffer without any chemotactic compound). Aspartate was used as the positive control and *o*-nitrophenol (ONP) and *p*-nitroaniline (PNA) as the negative controls, since ONP and PNA were shown not to induce chemotaxis in strain SJ98 in our previous studies [20].

#### Competitive capillary assay

Two capillaries individually filled with chemotaxis buffer containing the optimal chemotactic concentration of either the test CNAC or a competitor attractant (either NACs such as PNP, 4-NC or ONB/PNB or aspartate) were immersed together in a suspension of strain SJ98 cells (10^7-8 ^cells.ml^-1^) and incubated at ambient temperature for 30 min. A third capillary filled with assay buffer and separately immersed in an induced SJ98 cell suspension was used as the negative control. CI values for test capillaries were then determined as described above.

### Chemicals

All the CNACs and putative intermediates were obtained from Sigma Aldrich (GmbH, Germany). Bacto agar was purchased from Difco laboratories (Detroit, USA), ferric ammonium sulphate and mercuric thiocyanate from Fluka Chemicals (Buchs, Switzerland) and *N*-(1-naphthyl)-ethylenediamine dyhydrochloride, sulphanilic acid, nitric acid and all the high quality growth media from local vendors. Calibrated capillary tubes (10 μl) used for capillary assays were procured from Drummond Scientific (Broomall, PA, USA). HPLC grade methanol, glacial acetic acid, trifluoroacetic acid and other solvents were obtained from Merck Limited (Darmstadt, Germany). All other chemicals and media used were of the highest purity grade.

## Results

### Metabolic activity of strain SJ98 on CNACs

Results obtained from an initial screening for metabolic activity of strain SJ98 on six test CNACs demonstrated that it could mineralize 2C4NP, 4C2NB and 5C2NB, whereas 2C3NP and 2C4NB could only be co-metabolically transformed in the presence of an alternative carbon source, and no metabolic activity was observed with 4C2NP (Additional File: Figures S1, S2). To determine whether the metabolized CNACs are transformed oxidatively or reductively, culture supernatants from transformation medium (MM + 10 mM sodium succinate plus test CNAC) were analyzed for the presence of nitrite or ammonia, respectively. 2C4NP and 2C3NP were oxidatively transformed, as determined by the presence of nitrite in culture supernatants, as was one of the three chloronitrobenzoates (CNBs) tested (2C4NB). The other two CNBs (4C2NB and 5C2NB) were transformed reductively, as indicated by the presence of ammonium in the culture medium. Culture supernatants collected from all of the transformed CNACs also tested positive for the presence of released Cl^- ^ions.

### Identification of transformation intermediates

Preliminary TLC studies of culture supernatants showed formation of *p-*nitrophenol (PNP), 4-nitrocatechol (4NC) and 1,2,4-benzenetriol (BT) from 2C4NP; identification of these metabolites was in agreement with our earlier report on SJ98-mediated degradation of 2C4NP [19]. Metabolites identified from the metabolic activity of strain SJ98 on other tested CNACs were as follows: *m*-nitrophenol (MNP) and 3-nitrocatechol (3NC) from 2C3NP; *o-*nitrobenzoate (ONB) and 3-hydroxyanthranilate (3HAA) from 4C2NB and 5C2NB; and *p-*nitrobenzene (PNB) and 3,4-dihydroxybenzoic acid (34DHBA) from 2C4NB. GC and HPLC analyses using authentic standards confirmed the identity of these intermediates (Table 1). No metabolite could be detected for 4C2NP with any of the chromatographic methods used.

**Table 1:** Identification of metabolites formed during transformation of different CNACs by strain SJ98

	GC Rt of substrates and metabolites (min)		HPLC Rt of substrates and metabolites (min)		Identified metabolites
	
	Substrate	Metabolite	Substrate	Metabolite	
**Test compounds**					

2C4NP	2.66	2.43, 4.18, 5.99	2.16	1.98, 3.58, 4.21	PNP, 4NC, BT

2C3NP	2.42	2.31	2.07	1.86,3.49	MNP, 3NC

4C2NP	2.24	ND	2.03	ND	ND

2C4NB	2.74	2.1, 3.60	19.45	3.53	PNB, 3,4DHBA

4C2NB	2.51	2.88, 3.26	21.87	2.36, 3.89	ONB, 3HAA

5C2NB	2.52	2.875, 3.24	26.98	2.41, 3.92	ONB, 3HAA

**Standards**					

PNP	2.44		1.99		

4NC	4.17		3.59		

BT	5.94		4.19		

MNP	2.32		1.88		

3-Nitrocatechol	ND		3.50		

PNB	2.11		3.53		

3,4DHBA	3.60		ND		

ONB	2.88		2.36		

3HAA	3.25		3.91		

*ND *not determined; *PNP p-*nitrophenol, *4NC *4-nitrocatechol, *BT *benzenetriol, *MNP m*-nitrophenol, *3NC *3-nitrocatechol, *PNB p-*nitrobenzoate, *3,4DHBA *3,4- dihydrooxybenzoate, *ONB o*-nitrobenzoate, *3HAA *3-hydroxyanthranilic acid

### Chemotaxis of strain SJ98 towards CNACs

**Figure 1:**
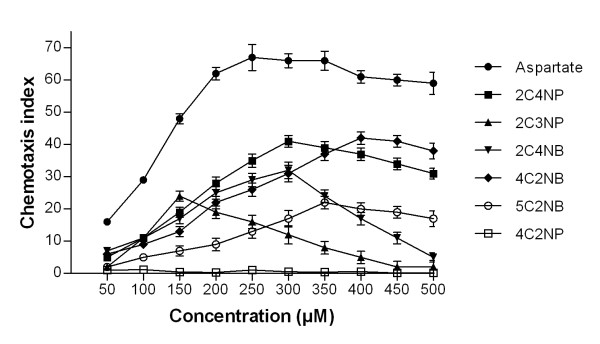
**Quantitation of the chemotactic response and determination of optimal response concentration for SJ98 chemotaxis towards different test compounds using capillary assays**. Values are presented as arithmetic means and error bars indicate standard deviations based on three independent replicate experiments.

Strain SJ98 was tested for chemotaxis towards all six CNACs by quantitative as well as qualitative assays. A primary screen with a capillary chemotaxis assay indicated concentration-dependent chemotaxis and semi bell-shaped concentration response curves for all CNACs except 4C2NP. As shown in Figure 1, the CI values for the other five compounds gradually increased with increasing concentrations of CNACs up until the optimal concentrations. Further increases in concentration led to sharp declines for 2C3NP and 2C4NB or plateaus for 2C4NP, 4C2NB and 5C2NB in the strength of the chemotactic response. The optimal chemotactic response concentrations were in the range 150-400 μM for all the tested CNACs except 4C2NP where no response was observed at any concentration. Significantly, 4C2NP was also the compound for which no metabolism had been observed. The strongest chemotactic response was observed for 2C4NP and 4C2NB, with CI values of 41 and 42, respectively, at their respective optimal response concentrations (Figure 1). Interestingly, these two chemoattractants were both mineralized whereas the third mineralized chemoattractant, 5C2NB, only gave a modest CI of 22.

**Figure 2:**
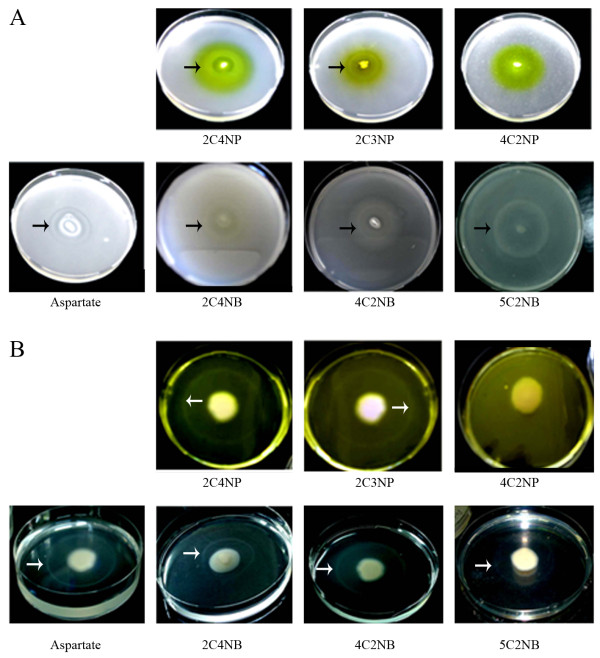
**Chemotaxis of *Burkholderia *sp. strain SJ98 towards different CNACs monitored with (**A**) drop plate assays; and (**B**) swarm plate assays**. Cells of strain SJ98 were grown in the presence of the respective CNAC and then tested for chemotaxis. Both the assays were preformed in triplicate and the representative plates are shown here. Aspartate was used as the positive control. Positive chemotaxis was determined by monitoring the formation of bacterial cell accumulation in the form of concentric chemotactic rings.

Results from qualitative drop plate and swarm plate chemotaxis assays validated the findings of the capillary assays; positive chemotaxis (determined by the formation of bacterial migration rings) could be observed for all five CNACs that were metabolically transformed by strain SJ98, but not for 4C2NP (Figure 2).

### Inducibility of SJ98 chemotaxis towards CNACs

**Figure 3:**
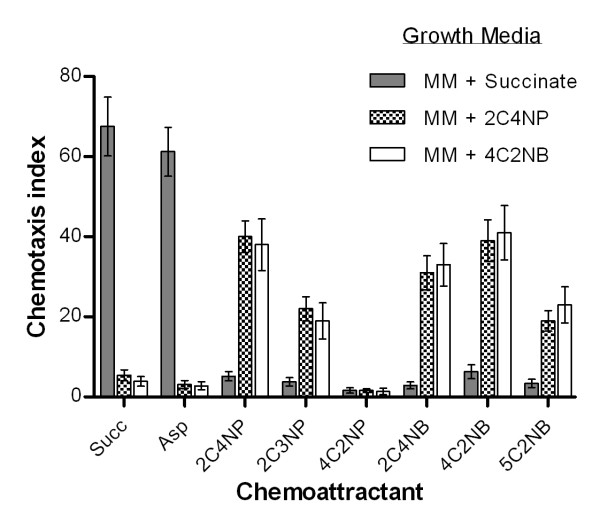
**Effect of growth substrate/metabolic induction on the chemotactic response of *Burkholderia *sp. strain SJ98 towards optimal concentrations of CNACs**. Cells of strain SJ98 were grown on succinate or a CNAC at its optimal response concentration as the sole source of carbon and energy and subsequently subjected to chemotaxis. Values are presented as arithmetic means and error bars indicate standard deviations based on three independent replicates.

Quantitative capillary chemotaxis assays were then performed with cells of strain SJ98 grown in (i) MM plus 10 mM succinate; (ii) MM + 300 μM 2C4NP and (iii) MM + 300 μM 4C2NB. 2C4NP and 4C2NB were chosen for the latter two induction conditions because their nitro groups were oxidatively vs. reductively transformed by strain SJ98, respectively. Cells grown in the absence of 2C4NP or 4C2NB exhibited much weaker chemotactic responses towards all five CNACs testing positive in the assays above than did those grown in the presence of the CNACs (Figure 3). There were no major difference in the strength of the effects of growth on the two CNACs and there was essentially no effect of growth on succinate, albeit the latter did strongly induce chemotaxis towards succinate or aspartate. The inductive effect of growth on the two CNACs was most noticeable for 2C4NP and 4C2NB, for which the CI values dropped by 91% and 87%, respectively; CI values decreased by 60-80% for the other three CNACs eliciting chemotactic responses (Figure 3).

### SJ98 chemotaxis towards CNACs in the presence of competitive chemoattractants

**Figure 4:**
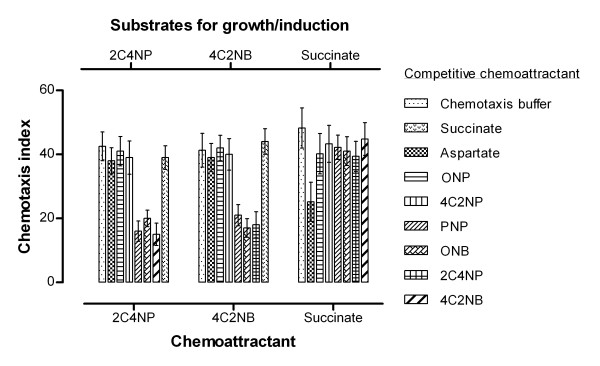
**Chemotaxis of *Burkholderia *sp. strain SJ98 towards 2C4NP, 4C2NB and succinate in the presence of other chemicals as competitive attractant**. Cells of strain SJ98 grown on 2C4NP, 4C2NB or succinate were subjected to capillary assays in the presence of a second capillary filled with a test chemical (shown in the figure). Values are presented as arithmetic means and error bars indicate standard deviations based on three independent replicates.

Competitive capillary chemotaxis assays were performed to test how the chemotaxis of strain SJ98 towards CNACs is affected by the presence of another chemoattractant. In previous studies, strain SJ98 was reported to be chemotactic towards a number of NACs and simple carbon sources e.g. succinate, aspartate etc. [20-22]. We therefore used capillaries containing optimal response concentrations of different NACs, aspartate or succinate as competitive chemoattractants. Cells of strain SJ98 grown on 2C4NP or 4C2NB as the sole source of carbon and therefore induced for chemotaxis towards CNACs were used for the assays. Results from these experiments showed ~40-55% lower CI values in the presence of a NAC known to be a chemoattractant (PNP, 4-NC or ONB) (Figure 4). However no decrease in chemotactic response was observed in the presence of either aspartate or succinate. Significantly, the presence of 4C2NP or *o*- nitrophenol (ONP) (a CNAC and a NAC that are not transformed by strain SJ98; see above and [20]) did not elicit an inhibitory effect (Figure 4).

This assay was then repeated with cells grown on succinate as the sole carbon source. Notably, these cells showed no chemotaxis towards CNACs and the presence of CNACs as the competitor did not reduce their chemotaxis towards succinate. By contrast, aspartate competitively inhibited their chemotaxis towards succinate (Figure 4). Together, these results indicate that strain SJ98 exhibits differentially inducible chemotaxis towards different groups of molecules. This observation also suggests the possibility that different chemo-receptors detect the presence/metabolism of different chemoattractants. Further studies are required to decipher the molecular mechanism(s) for such differential induction of chemotactic responses.

## Discussion

Microbial chemotaxis has recently been proposed as a widespread phenomenon among motile bacteria towards several distinct xenobiotic compounds and it may therefore be advantageous to use such bacteria in bioremediation [31]. It is suggested that chemotaxis can enhance biodegradation by effectively improving 'pollutant bioavailability' and/or by promoting the formation of microbial consortia with diverse microorganisms harboring complementary degradation capabilities [7,8,31,32]. Several studies have now reported the isolation and characterization of bacteria responding chemotactically to a wide variety of hazardous environmental pollutants, including toluene, trinitrotoluene, atrazine and a variety of nitroaromatic compounds [7-9,33]. However, information pertaining to bacterial chemotaxis towards some of the recently introduced, highly recalcitrant, chlorinated xenobiotic compounds (e.g. chloro-nitroaromatic compounds, polychlorinated biphenyls, chlorinated anilines etc.) is extremely scarce [31].

Results presented in this report clearly demonstrate that *Burkholderia *sp. strain SJ98 is chemotactic towards five CNACs. Furthermore, there is a strong association between the chemotaxis and metabolic transformation of the compounds; a chemotactic response was only observed towards those CNACs that the strain could either completely degrade or co-metabolically transform in the presence of alternative carbon sources. Based on observed intermediates, the following catabolic pathways are proposed for CNACs degradation in strain SJ98: (1) both 4C2NB and 5C2NB are degraded via ONB and 3HAA; (2) 2C4NB is transformed to 3,4DHBA via PNB; and (3) 2C3NP is transformed to 3NC via MNP. The degradation pathway for 2C4NP is via PNP, 4NC and BT, as has already been reported [25]. Interestingly, some of the intermediates identified from the five chemoattractant CNACs degradation/transformation were previously characterized chemoattractants for strain SJ98. These are (1) PNP and 4NC in the 2C4NP pathway; (2) ONB in the 4C2NB and 5C2NB pathways; [3] PNB in the 2C4NB pathway; and (4) MNP in the 2C3NP pathway. These pathways and chemotactic intermediates have been summarized in Additional file: Figure S3. Chemotaxis of strain SJ98 towards 2C4NP, 4C2NB and 5C2NB and also towards some of their metabolic intermediates strongly suggests metabolism depended chemotaxis to this strains towards these CNACs.

Previous studies have suggested two mechanisms for bacterial chemotaxis towards xenobiotic compounds [8]. The first involves transmembrane signaling by a bacterial chemoreceptor wherein binding of the ligand to the extracellular domain of the chemoreceptor generates a transducible signal and results in chemotaxis. This mechanism is independent of metabolism of the chemoattractant and can therefore also be induced by non-metabolizable structural analogues of the chemoattractant. The second possible mechanism involves energy flux, wherein changes in cellular energy levels resulting from metabolism of chemoattractant molecules induce the chemotactic response. It is necessary for the chemoattractant to be metabolized for this mechanism to be operative [34]. Empirical work on various systems to date provides support for both mechanisms. In support of the first mechanism, Liu and Parales recently reported that *Pseudomonas *sp. strain ADP was chemotactic towards both atrazine, which it could metabolise, and its *s*-triazine analogue ametryn, which it could not [35]. They also showed that atrazine degradation and chemotaxis are genetically distinct phenotypes in strain ADP. By contrast, support for the second mechanism comes from studies of the chemotaxis by *Pseudomonas putida *G7 towards naphthalene [6,36], *P. putida *F1 towards toluene [9], and *Ralstonia eutropha *JMP134 towards 2,4-dichlorophenoxyacetate [37], which have all reported the phenomenon to be dependent on and genetically linked to the metabolism of the chemoattractant. It remains to be determined whether the proximal triggers for the chemotactic response are the CNACs themselves or their, e.g. NAC, metabolites.

Our results suggest that a more complex mechanism may operate in respect of the chemotaxis of strain SJ98 towards CNACs. The fact that strain SJ98 does not show chemotaxis towards the non-metabolizable structural analogue 4C2NP suggests metabolism-dependent effects. However, the ability of strain SJ98 to be attracted towards co-metabolically transformed NACs [17] and CNACs is a notable departure from previous examples of metabolism-dependent mechanisms and raises questions as to the extent of energy flux needed for metabolism-dependent chemotaxis.

Also significant is our finding that cells of strain SJ98 induced to metabolise CNACs can exhibit selective chemotaxis towards CNACs which is not inhibited by co-occurrence of simpler compounds like aspartate or succinate as alternative chemoattractants. This finding confirms that CNAC chemotaxis by this strain is at least to some degree a separate phenomenon from some of the precedents. This could also be an important advantage in the potential application of this strain in the *in situ *bioremediation of CNAC-contaminated sites. Specific regulation of chemotaxis towards the target compound in contaminated environments often comprising a complex mix of multiple potential chemoattractants could significantly improve the efficiency of *in situ *bioremediation. The chemotaxis of strain SJ98 towards CNACs therefore could be a fruitful model system for studying both basic and applied aspects of target-specific bacterial chemotaxis.

## Conclusions

*Burkholderia *sp. strain SJ98 exhibits chemotaxis to five CNACs which can either be mineralized (2C4NP, 4C2NB and 5C2NB) or co-metabolically transformed (2C3NP and 2C4NB) by it. On the other hand no chemotaxis was observed towards 4C2NP which was not metabolized by this strain. This chemotaxis towards metabolizable CNACs appears to be related to that previously shown for NACs that are metabolized by this strain but it is induced independently of the chemotaxis which this strain shows towards succinate and aspartate.

## Abbreviations

CNACs: chloro-nitroaromatic compounds; 2C4NP: 2-chloro-4-nitrophenol; 2C3NP: 2-chloro-3-nitrophenol; 4C2NP: 4-chloro-2-nitrophenol; 2C4NB: 2-chloro-4-nitrobenzoate; 4C2NB: 4-chloro-2-nitrobenzoate; 5C2NB: 5-chloro-2-nitrobenzoate; CNPs: chloronitrophenols; CNBs: chloronitrobenzoates; PNP: p-nitrophenol; 4NC: 4-nitrocatechol; BT: 1,2,3-benzenetriol; MNP: m-nitrophenol; ONP: o-nitrophenol; 3NC: 3-nitrocatechol; PNA: p-nitroaniline; PNB: p-nitrobenzoate; 3,4DHBA: 3,4-dihydroxybenzoic acid; ONB: o-nitrobenzoate; 3HAA: 3-hydroxyanthranilic acid; CI: chemotaxis index.

## Authors' contributions

JP, NKS, RKJ and GP conceived the idea and designed the experiments. JP, NKS, FK and AG carried out the experiments. JP, JGO and GP prepared the manuscript. All authors except RKJ have read and approved the final manuscript.

## Authors' information

The other authors wish to acknowledge the inspiration of RKJ who fell ill early in the conduct of the work and passed away before the manuscript was ready for communication.

## Supplementary Material

Additional file 1 **Figure S1**. (**A**) Growth of strain SJ98 on 300 μM CNACs as sole source of carbon and energy, and (**B**) Degradation of CNACs by strain SJ98 as a sole source of carbon and energy. **Figure S2**. Degradation of CNACs by induced resting cells of strain SJ98. **Figure S3**. Catabolic pathways for degradation of five chemoattractant CNACs which are either mineralized (2C4NP, 4C2NP and 5C2NB) or co-metabolically transformed (2C4NB and 2C3NP) by strain SJ98. Metabolites marked with asterisk (PNP, 4NC, ONB, PNB and MNP) have also been previously reported as chemoattractants for this strain (19-22). Click here for file
