# Supplementary material for: Chemotaxis of Burkholderia sp. Strain SJ98 towards chloronitroaromatic compounds that it can metabolise
Source: BMC Microbiol. 2012 Feb 1;12:19. doi: 10.1186/1471-2180-12-19 (PMC3293717; doi:10.1186/1471-2180-12-19)
Supplement: Additional file 1 — Figure S1. (A) Growth of strain SJ98 on 300 μM CNACs as sole source of carbon and energy, and (B) Degradation of CNACs by strain SJ98 as a sole source of carbon and energy. Figure S2. Degradation of CNACs by induced resting cells of strain SJ98. Figure S3. Catabolic pathways for degradation of five chemoattractant CNACs which are either mineralized (2C4NP, 4C2NP and 5C2NB) or co-metabolically transformed (2C4NB and 2C3NP) by strain SJ98. Metabolites marked with asterisk (PNP, 4NC, ONB, PNB and MNP) have also been previously reported as chemoattractants for this strain (19-22). [file 1471-2180-12-19-S1.DOC]

**Additional File**


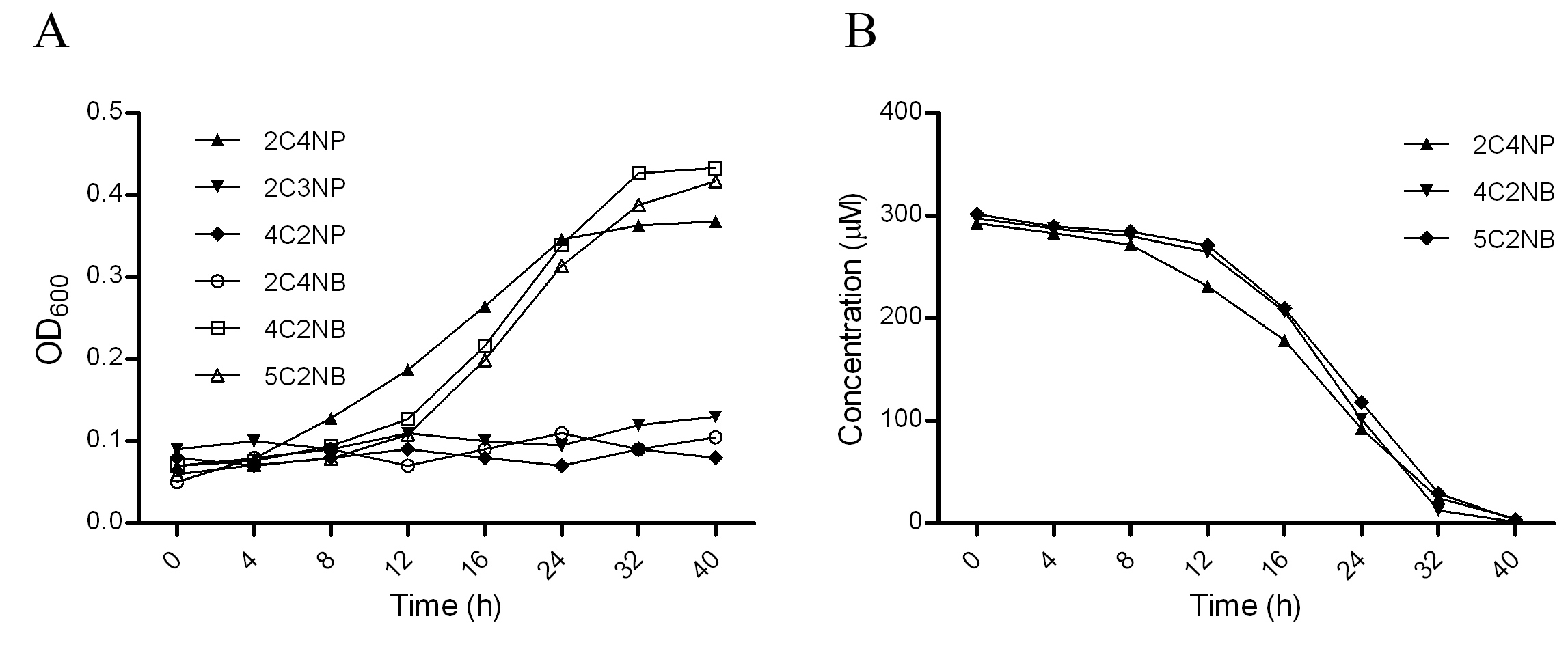


**Figure S1.** (A) Growth of strain SJ98 on 300 µM CNACs as sole source of carbon and energy, and (B) Degradation of CNACs by strain SJ98 as a sole source of carbon and energy.


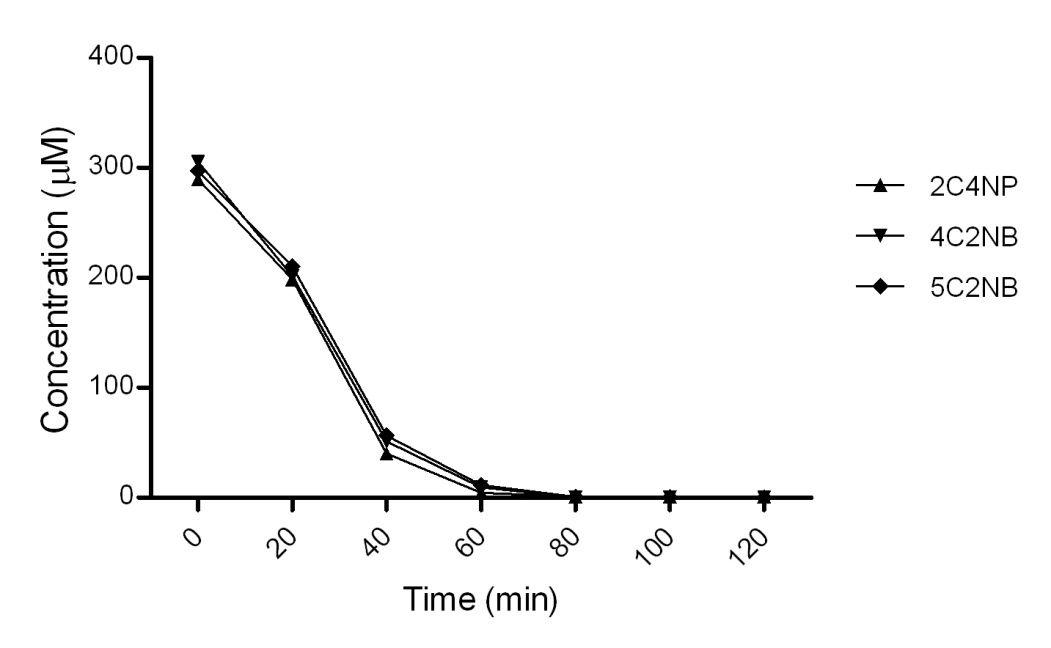


**Figure S2.** Degradation of CNACs by induced resting cells of strain SJ98.

**Figure S3.** Catabolic pathways for degradation of five chemoattractant CNACs which are either mineralized (2C4NP, 4C2NP and 5C2NB) or co-metabolically transformed (2C4NB and 2C3NP) by strain SJ98. Metabolites marked with asterisk (PNP, 4NC, ONB, PNB and MNP) have also been previously reported as chemoattractants for this strain (19-22).
